# Efficacy of mindfulness-based stress reduction versus usual care in patients with breast cancer: a meta-analysis with trial sequential analysis of randomized controlled trials

**DOI:** 10.3389/fonc.2026.1780224

**Published:** 2026-07-29

**Authors:** Jing Lei, Meimei He

**Affiliations:** 1 Teaching and Research Section of Clinical Nursing, The Second Xiangya Hospital, Central South University, Changsha, China; 2 Department of Orthopedics, The Second Xiangya Hospital, Central South University, Changsha, China; 3 Department of Hematology, The Second Xiangya Hospital, Central South University, Changsha, China

**Keywords:** anxiety, breast cancer, depression, MBSR, mindfulness-based stress reduction, quality of life

## Abstract

**Background:**

Mindfulness-based stress reduction (MBSR) has shown promise in improving physical and mental health in breast cancer patients, warranting further comprehensive evaluation. This meta-analysis aimed to assess the effects of MBSR compared to usual care on the physical and psychological well-being of patients with breast cancer.

**Methods:**

A systematic search of Web of Science, PubMed, Cochrane Library, and Embase was conducted to identify RCTs published up to October 5, 2025. Eligible studies enrolled patients with breast cancer at any stage, compared MBSR with usual care or waitlist control, and reported psychological, symptom-related, or quality of life (QoL) outcomes. Non-randomized studies, single-arm trials, duplicate reports, studies without relevant outcomes or sufficient data, and reviews, case reports, conference abstracts, letters, and animal studies were excluded. The primary outcomes were anxiety, depression, fatigue, and overall QoL. Standardized mean differences (SMDs) or mean differences (MDs) with 95% confidence intervals (CIs) were pooled according to outcome measurement scales. Heterogeneity was assessed using I^2^, Cochran’s Q test, and 95% prediction intervals (PIs); fixed- or random-effects models were selected according to heterogeneity. Subgroup, sensitivity, and publication bias analyses were performed. Trial sequential analysis was conducted using TSA v0.9.5.10 Beta. The protocol was registered in PROSPERO (CRD420261277936).

**Results:**

This meta-analysis included 20 studies comprising 2534 breast cancer patients. The overall results indicated that compared with usual care, MBSR significantly alleviated anxiety (SMD: -0.489, 95% CI: -0.734 to -0.244, 95% PI: -1.520 to 0.541), depression (SMD: -0.258, 95% CI: -0.347 to -0.170, 95% PI: -0.650 to 0.083), and fatigue (SMD: -0.399, 95% CI: -0.562 to -0.236, 95% PI: -0.963 to 0.166), while markedly improving overall QoL (SMD: 0.262, 95% CI: 0.059 to 0.465, 95% PI: -0.311 to 0.836). Additional benefits of MBSR included reductions in perceived stress and pain, improvements in posttraumatic growth, and decreases in total mood disturbance (all *p* < 0.05). Further subgroup analyses revealed that the 8-week MBSR protocol yielded more psychological and QoL improvements compared to the 6-week program. Notably, the benefits of MBSR in reducing depression, fatigue, perceived stress, and promoting posttraumatic growth remained observable at 4 to 9 weeks after the intervention (all *p* < 0.05), emphasizing its sustained efficacy.

**Conclusion:**

Our findings support MBSR as an effective intervention for improving the psychological health and QoL of breast cancer patients. Future research should focus on the long-term efficacy of MBSR in this population.

**Systematic review registration:**

https://www.crd.york.ac.uk/PROSPERO/, identifier CRD420261277936.

## 1 Introduction

Breast cancer, now recognized as the second most frequently diagnosed malignancy worldwide after lung cancer, is the leading cause of cancer-related morbidity and mortality among women, as reported by the Global Cancer Statistics 2022 (1). Despite progress in early diagnosis and therapeutic strategies, the prognosis for the overall physical and mental well-being of individuals diagnosed with breast cancer remains concerning (2, 3). Compared to the general population and patients with other malignancies, individuals with breast cancer are more likely to experience heightened levels of anxiety, depression, and sleep disturbances (4–6). These psychological impairments often result in reduced quality of life (QoL), prolonged hospitalization, and poorer compliance with medical treatments (4–6). The considerable psychological burden and associated adverse outcomes linked to breast cancer underscore the critical need for the development of targeted interventions aimed at enhancing both physical and mental health, as well as improving QoL in affected patients.

Rehabilitation has become an increasingly important element of survivorship care for individuals with breast cancer. Guided by the International Classification of Functioning, Disability and Health, rehabilitation extends beyond the management of bodily impairments to include difficulties in daily activities, social participation, and reintegration into community life (7). Following treatment for breast cancer, many patients report fatigue, pain, diminished physical capacity, weight-associated functional restrictions, and emotional distress, which can hinder recovery and adversely affect QoL (8). Findings from studies of exercise-based rehabilitation also indicate that structured programmes can enhance functional recovery after surgery (9). Therefore, an integrated rehabilitation approach should support both physical recovery and psychological adjustment during the post-treatment period.

Various approaches have been implemented to address the physical and psychological challenges faced by breast cancer patients undergoing chemotherapy (10–12). Among these, mindfulness-based stress reduction (MBSR), developed by Professor Jon Kabat-Zinn at the University of Massachusetts Medical School, emphasizes cultivating present-moment awareness with openness, curiosity, and acceptance (13). This mindfulness-based intervention incorporates key practices such as body scanning, mindful breathing, walking meditation, psychoeducation, and mindful movement, all aimed at mitigating pain and alleviating stress (14). Empirical evidence from studies in breast cancer populations has suggested that MBSR contributes to improved mental health outcomes, enhanced female sexual function (15), and increased posttraumatic growth (16), while also reducing psychological symptoms (17). A 2019 systematic review of randomized clinical trials evaluating MBSR in women with breast cancer concluded that the intervention provides modest improvements in fatigue, anxiety, and sleep quality, as well as a moderate reduction in depressive symptoms over the short term (18).

Accumulating research has underscored the therapeutic potential of MBSR in alleviating psychological distress, including anxiety and depression, while enhancing QoL in individuals with breast cancer (19, 20). Recent systematic reviews further affirm the role of MBSR in improving both physical and mental health outcomes in this population (21, 22). In light of newly published randomized controlled trials (RCTs) presenting updated findings on MBSR intervention for breast cancer (23–25), alongside the limitations of prior meta-analysis that has yet to comprehensively evaluate outcomes encompassing all aspects of patients’ physical and mental health (26), we conducted a comprehensive meta-analysis incorporating the latest RCTs to assess the effect of MBSR on anxiety, depression, overall QoL, and various QoL indicators compared with usual care in breast cancer patients. Furthermore, subgroup analyses were conducted to investigate the impact of varying intervention durations and post-treatment assessment intervals on measured outcomes. Through this thorough evaluation of existing literature, our study aimed to deepen understanding, strengthen the evidence base, and provide valuable guidance for integrating psychological care into the clinical management of breast cancer.

## 2 Methods

### 2.1 Study design

The research adhered rigorously to the guidelines outlined by the Preferred Reporting Items for Systematic Reviews and Meta-Analyses (PRISMA) (27). Additionally, the study protocol was prospectively recorded in the PROSPERO database for systematic review evaluations under the registration identifier CRD420261277936.

### 2.2 Literature search strategy

Two researchers conducted independent and systematic searches across the Web of Science, PubMed, Cochrane Library, and Embase databases, covering all records from their inception to October 5, 2025. The search strategy utilized a combination of standardized subject headings and free-text keywords, customized to align with the indexing protocols of each database. Key search terms included combinations such as (“mindfulness-based stress reduction” OR “MBSR” OR “mindfulness-based intervention”) AND (“breast cancer” OR “breast neoplasm” OR “breast tumor” OR “cancer of breast” OR “mammary cancer” OR “breast carcinoma”) AND (“randomized controlled trial” OR “controlled clinical trial” OR “randomized”). The detailed search strategy is provided in **Supplementary Files 1**. The search focused exclusively on studies involving human participants and imposed no restrictions on language. Additionally, reference lists from relevant systematic reviews and meta-analyses were manually screened to identify any further eligible studies.

### 2.3 Study selection

Eligible studies were identified based on the following criteria (1): RCTs (2); participants diagnosed with breast cancer at any stage (3); the experimental group received MBSR, which may include practices such as meditation, body scanning, mindfulness yoga, and zazen (4); the comparison group underwent usual care or was assigned to a waitlist (5); outcomes assessed included psychological factors (e.g., anxiety, depression, fatigue), overall QoL, and specific indicators such as perceived stress, pain, mental health, posttraumatic growth, fear of recurrence, general health, total mood disturbance, and sleep quality. Studies were excluded if they met any of the following criteria (1): non-randomized designs, observational studies, or single-arm trials (2); insufficient data or absence of relevant outcome measures (3); duplicate publications (4); case reports, animal studies, systematic reviews, meta-analyses, conference abstracts, or letters. Study selection was conducted according to predefined eligibility criteria. After duplicates were removed, two reviewers independently screened titles and abstracts and then assessed the full texts of potentially eligible articles. Disagreements at any stage were resolved through discussion or, when consensus could not be reached, by consultation with a third reviewer.

### 2.4 Data extraction and quality assessment

In this research, two reviewers independently extracted data using a predefined data extraction form, and any discrepancies were resolved through discussion until consensus was reached. The collected data encompassed the name of the first author, publication year, study design, country, characteristics of the study population, sample size, participant age, intervention and control details, time point for outcome assessment, and reported outcomes. For trials with multiple publications, the most comprehensive and detailed report was prioritized for inclusion. The primary outcomes analyzed were anxiety, depression, fatigue, and overall QoL, while secondary outcomes included specific QoL measures, such as perceived stress, pain, mental health, posttraumatic growth, fear of recurrence, general health, total mood disturbance, and sleep quality.

The risk of bias in the included studies was appraised independently by two reviewers using the modified Jadad scale (28), which considers randomization procedures, allocation concealment, blinding, and reporting of participant attrition. Studies were scored on a 7-point scale, with ratings of 1–3 indicating low quality and 4–7 indicating high quality (29). Any disagreements between reviewers were addressed through discussion or, if necessary, resolved by consulting a third reviewer to reach a consensus.

### 2.5 Statistical analysis

In the analyzed studies, all outcomes were presented as continuous variables, expressed through means and standard deviations. To standardize effect sizes for outcomes measured with varying methods or scales, standardized mean differences (SMDs) with 95% confidence intervals (CIs) were calculated. For outcomes using identical measurement tools, mean differences (MDs) with corresponding 95% CIs were applied. Heterogeneity across studies was quantified using the I^2^ statistic, Cochrane Q test, and 95% prediction intervals (PIs) (30, 31). A fixed-effects model was employed when heterogeneity was low (*p* ≥ 0.10 or I^2^ ≤ 50%), while a random-effects model was adopted in cases of significant heterogeneity (32). Subgroup analyses were conducted to assess the influence of intervention duration (6 weeks vs. 8 weeks) and the timing of outcome evaluation (immediately post-intervention vs. 4–9 weeks post-intervention) on the efficacy of MBSR. Sensitivity analyses were undertaken by sequentially excluding individual studies to examine the stability of pooled estimates. Publication bias was evaluated using funnel plots, the trim-and-fill method, and Begg’s and Egger’s tests (33, 34). All statistical analyses were carried out using R software 4.3.2 and STATA 12.0, with statistical significance set at *p* < 0.05.

### 2.6 Trial sequential analysis

Trial sequential analysis (TSA) was employed in the meta-analysis to control the risks of type I and type II errors (35). Continuous outcomes were analyzed using TSA v0.9.5.10 Beta software, which estimated the required information size (RIS) and delineated trial sequential monitoring boundaries. When the cumulative Z-curve crossed the RIS or monitoring thresholds, the evidence was deemed sufficient, indicating that further studies were unlikely to alter the conclusions. The RIS calculation was based on predefined criteria, including a two-sided significance level (α = 0.05) and statistical power (1-β = 0.80).

## 3 Results

### 3.1 Literature search results

**Figure 1:**
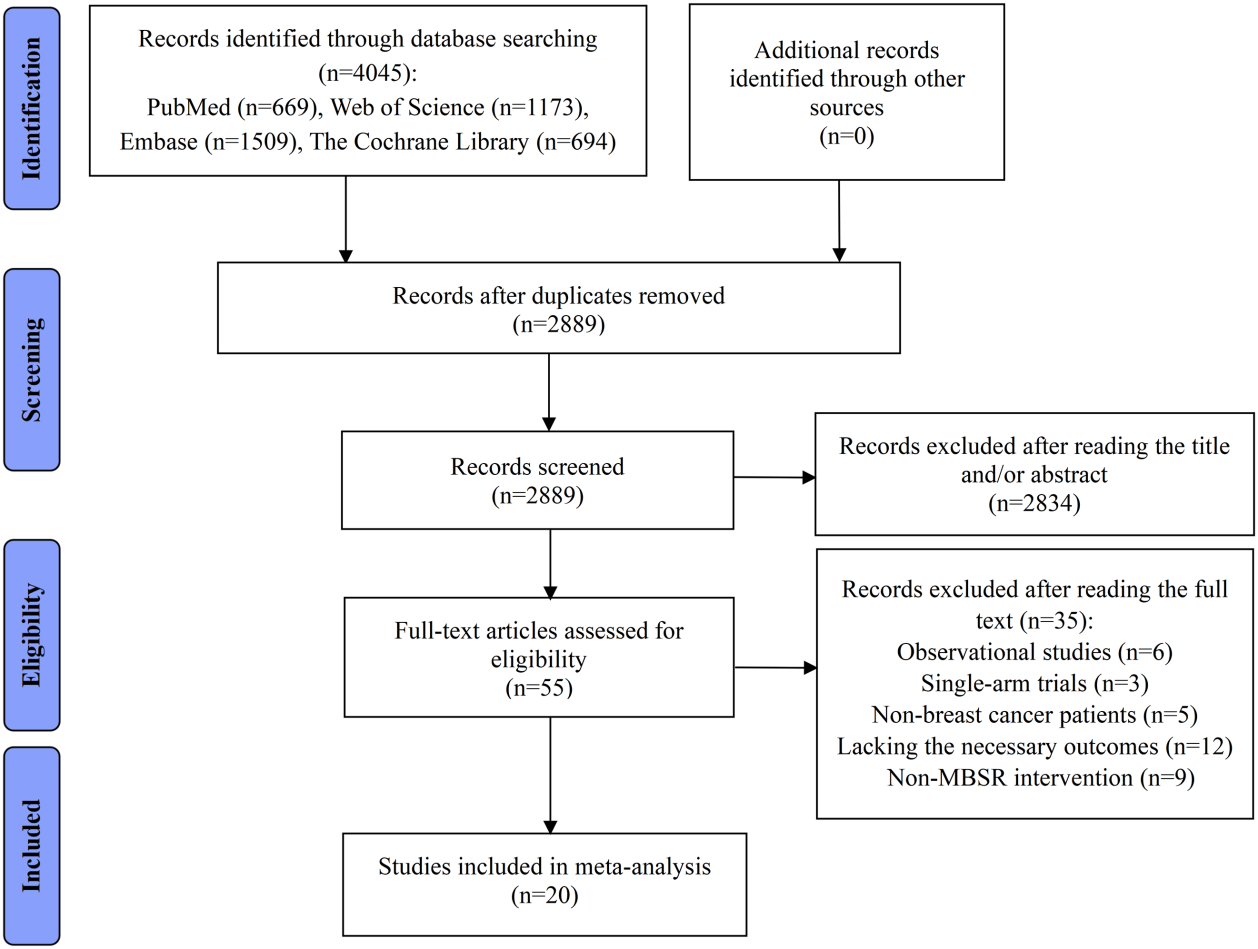
Flow diagram of the process of study selection.

The process of selecting RCTs for inclusion in the meta-analysis is depicted in **Figure 1**. The initial database search identified 4045 records, which were reduced to 2889 after removing duplicates. Titles and abstracts were screened, resulting in the exclusion of 2834 irrelevant articles. This process left 55 studies for full-text review. Of these, 35 were excluded for the following reasons: 6 were observational studies, 3 were single-arm trials, 5 investigated non-breast cancer populations, 12 did not report the required outcomes, and 9 did not employ MBSR as the intervention. Finally, 20 studies fulfilled the inclusion criteria and were incorporated into the meta-analysis (16, 23–25, 36–51).

### 3.2 Study characteristics and quality assessment

The study characteristics are presented in **Table 1**. This meta-analysis incorporated a total of 20 studies, all of which were RCTs, comprising 2534 participants. Of these, 1339 individuals with breast cancer received MBSR, while 1195 were allocated to the control group receiving usual care. Two distinct formats of MBSR were employed: the original 8-week program developed by Kabat-Zinn (52, 53) and a modified 6-week version designed by Dr. Lengacher (41) to better accommodate the needs of breast cancer patients. Outcome assessments were conducted at two time points: immediately following the intervention and during follow-up periods (e.g., at 2 months post-intervention). The tools and scales employed for outcome evaluation are outlined in **Supplementary Table 1**. MBSR intervention components and delivery methods in the included studies are detailed in **Supplementary Table 2**. Quality appraisal identified 17 studies as high-quality, while 3 were classified as low-quality, primarily due to limitations in achieving double blinding. Detailed information on the quality assessment can be found in **Supplementary Table 3**.

**Table 1:** Basic characteristics of the included studies.

Author (year)	Study design	Country	Participants	Sample size (I/C)	Age (I/C, years)	Intervention group	Control group	Time point for outcome assessment	Outcomes
Zhu et al. (2023) (51)	RCT	China	Breast cancer patients who completed the first chemotherapy after surgical treatment	50/51	47.96 ± 8.51/49.78 ± 7.48	1 MBSR session a week over an 8-week period	Routine care without any MBSR intervention	Week 8	Anxiety, depression, and QoL
Mirmahmoodi et al. (2020) (46)	RCT	Iran	Breast cancer patients without metastasis and undergoing at least 1 chemotherapy period or surgery	22/22	44.14 ± 11.19/45.64 ± 10.11	MBSR in 90-minute sessions for 8 weeks (1 session a week)	Routine care, including consultations with psychiatric nurses and psychologists	Week 8	Anxiety, depression, and QoL
Kenne Sarenmalm et al. (2017) (39)	RCT	Sweden	Breast cancer patients after completion of adjuvant chemotherapy and/or radiation therapy, with or without endocrine therapy	62/52	57.2 ± 10.2	8 weeks self-instructing MBSR program + instructor and weekly group sessions	Standard care for breast cancer according to the national and local guidelines	Month 3	Anxiety, depression, and QoL
Lengacher et al. (2016) (43)	RCT	USA	Patients with a diagnosis of stage 0 to III breast cancer who had completed treatment	167/155	56.5 ± 10.2/57.6 ± 9.2	2-hour MBSR sessions once per week for 6 weeks	Usual care (standard post-treatment clinic visits)	Week 6 and 12	Anxiety, depression, fatigue, and QoL
Zhu et al. (2025) (25)	RCT	China	Breast cancer patients who completed the first chemotherapy after surgical treatment	50/51	47.96 ± 8.51/49.78 ± 7.48	1 MBSR session a week over an 8-week period	Routine care without any MBSR intervention	Week 8	QoL
Hoffman et al. (2012) (38)	RCT	UK	Women with stage 0 to III breast cancer who have completed surgery, chemotherapy, and/or radiotherapy	114/115	49.0 ± 9.26/50.1 ± 9.14	8-week MSBR program (8 weekly classes of 2 hours in length)	Usual care (without MBSR intervention)	Weeks 8 to 12 and weeks 12 to 14	Anxiety, depression, fatigue, and QoL
Shergill et al. (2022) (50)	RCT	Canada	Female breast cancer survivors with chronic neuropathic pain who were at least 1-year post-treatment of cancer	49/49	51.3 ± 11.4/55.1 ± 9.6	MBSR (including eight, 2.5-hour weekly sessions and a full day retreat held halfway through the course on a weekend)	Routine care (without MBSR intervention)	Week 2 and month 3	Depression and QoL
Lengacher et al. (2009) (41)	RCT	USA	Women with stage 0, I, II, or III breast cancer and who had completed treatment prior to study enrollment	41/43	57.5 ± 9.4	MBSR (weekly 2-hour sessions over a period of 6 weeks)	Usual care (without MBSR intervention)	Week 6	Anxiety, depression, and QoL
Henderson et al. (2013) (37)	RCT	USA	Women with newly diagnosed stage I or II cancer of the breast	53/58	20-65	MBSR (8 weekly 2.5- to 3.5-hour sessions with an additional 7.5-hour intensive retreat session given in the sixth week)	Usual care (excluding the MBSR)	Month 4	Anxiety
Lengacher et al. (2025) (24)	RCT	USA	Patients with stage I, II, or III breast cancer who completed treatment	91/31	56.3 ± 11.4/59.5 ± 11.0	MBSR (weekly 2-hour sessions over a period of 6 weeks)	Standard post-treatment care (without MBSR intervention)	Week 6, 12 and 26	Anxiety, depression, fatigue, and QoL
Zhang et al. (2017) (16)	RCT	China	Females with stage I-III breast cancer	30/30	48.67 ± 8.49/46.00 ± 5.12	MBSR, including 2-hour sessions weekly for a duration of 8 weeks	Usual care (without MBSR intervention)	Week 8 and month 3	Anxiety and QoL
Lengacher et al. (2012) (44)	RCT	USA	Women with stage 0, I, II, or III breast cancer and who had completed treatment prior to study enrollment	41/43	57.5 ± 9.4	MBSR (weekly 2-hour sessions over a period of 6 weeks)	Usual care (without MBSR intervention)	Week 6	Fatigue and QoL
Liu et al. (2022) (45)	RCT	China	Patients with breast cancer undergoing chemotherapy	38/34	48.58 ± 8.48/51.41 ± 10.10	MBSR practice once daily (lasting 45 min) for 8 weeks (each session for one week)	Routine care, including symptom assessment and management, usual psychological support, etc	Week 4 and 8	Anxiety, depression, fatigue, and QoL
Reich et al. (2017) (48)	RCT	USA	Patients with stage 0-III breast cancer and within two weeks to two years off treatment	167/155	56.5 ± 10.2/57.6 ± 9.2	2-hour MBSR sessions once per week for 6 weeks	Usual care (standard post-treatment clinic visits)	Week 6 and 12	Fatigue and QoL
Rahmani et al. (2015) (47)	RCT	Iran	Patients with stages I, II, or III of breast cancer	12/12	43.25 ± 3.07/44.08 ± 3.28	MBSR (once a week in a 2-hour session for 8 weeks)	Usual care	Week 8; and month 2 after MBSR	Fatigue and QoL
Lengacher et al. (2021) (40)	RCT	USA	Patients with a diagnosis of stage 0 to III breast cancer who had completed treatment	165/155	56 (mean age)	2-hour MBSR sessions once per week for 6 weeks	Usual care (standard post-treatment clinic visits without MBSR intervention)	Week 6 and 12	QoL
Carlson et al. (2013) (36)	RCT	Canada	Women with stage I, II, or III breast cancer who had completed treatments	113/54	54.66 ± 9.71/56.27 ± 10.89	MBSR (8 weekly group sessions of 90 minutes each plus a 6-hour workshop between weeks 6 and 7 for a total of 18 contact hours)	An approximation of usual care, including 1-day (6-hour) didactic stressmanagement seminar	Week 8	QoL
Pehlivan et al. (2025) (23)	RCT	Turkey	Breast cancer females who had undergone a modified radical mastectomy	19/20	44.84 ± 4.57/44.75 ± 4.40	MBSR (90-minute sessions per week for 8 weeks)	Routine care	Week 8; and month 2 after MBSR	QoL
Reich et al. (2014) (49)	RCT	USA	Female survivors with stage 0, I, II, or III breast cancer who had completed treatment	17/24	58.0 ± 10.3/58.2 ± 9.5	MBSR (2-hour sessions per week for 6 weeks)	Usual care (standard posttreatment clinic visits without MBSR intervention)	Week 6	Fatigue
Lengacher et al. (2015) (42)	RCT	USA	Women with stage 0-III breast cancer who had completed treatment prior to study enrollment	38/41	56.1 ± 9.1/58.0 ± 10.2	MBSR (2-h weekly sessions for 6 weeks)	Usual care (normal posttreatment clinic visits without MBSR intervention)	Week 6 and 12	QoL

I, intervention group; C, control group; RCT, randomized controlled trial; MBSR, mindfulness-based stress reduction; QoL, quality of life.

### 3.3 Meta-analysis of the primary outcomes

#### 3.3.1 Anxiety

**Figure 2:**
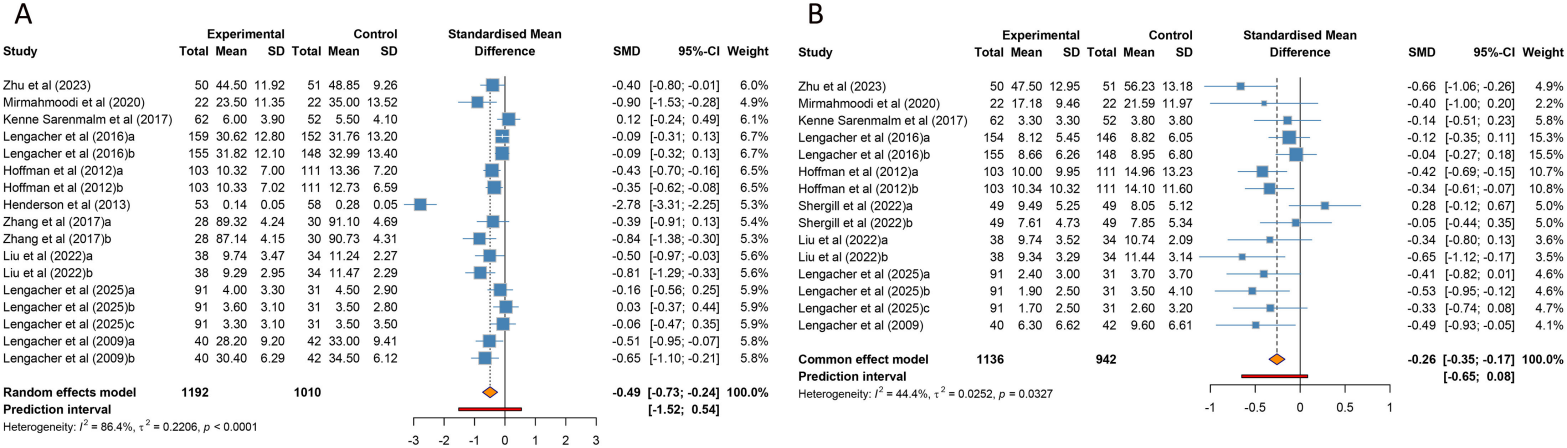
Forest plots of anxiety **(A)** and depression **(B)** after mindfulness-based stress reduction for breast cancer patients.

A total of 17 studies evaluated anxiety outcome. Due to substantial heterogeneity among the included studies (I^2^ = 86.4%, Tau^2^ = 0.221), a random-effects model was employed for analysis. The overall meta-analysis revealed that compared to the control group, MBSR significantly alleviated anxiety symptoms (SMD: -0.489, 95% CI: -0.734 to -0.244, 95% PI: -1.520 to 0.541) (**Table 2**; **Figure 2A**). Further subgroup analyses indicated that both the 6-week and 8-week MBSR interventions resulted in significant reductions in anxiety (all *p* < 0.05). Additionally, anxiety symptoms in the MBSR group showed significant improvements compared to the control group when assessed immediately post-intervention (SMD: -0.363, 95% CI: -0.485 to -0.241, 95% PI: -0.907 to 0.055). However, at 4 to 9 weeks post-intervention, no significant differences in anxiety levels were observed between the MBSR group and the control group (SMD: -0.542, 95% CI: -1.098 to 0.014, 95% PI: -2.440 to 1.355) (**Table 2**; **Supplementary Figure 1**).

**Table 2:** Pooled effect and subgroup analysis of the primary outcomes of mindfulness-based stress reduction for breast cancer.

Outcomes and subgroups	Number of studies	Meta-analysis				Heterogeneity	
		SMD	95% CI	p value	95% PI	I^2^, Tau^2^	p value
Anxiety							
Overall	17	-0.489	-0.734, -0.244	<0.001	-1.520, 0.541	86.4%, 0.221	<0.001
Treatment duration							
6 weeks	7	-0.156	-0.277, -0.035	0.012	-0.532, 0.178	33.5%, 0.015	0.172
8 weeks	10	-0.709	-1.118, -0.299	0.001	-2.185, 0.768	89.9%, 0.382	<0.001
Time points of outcome measurement							
Immediately post-intervention	9	-0.363	-0.485, -0.241	<0.001	-0.907, 0.055	48.2%, 0.035	0.051
4-9weeks post-intervention	7	-0.542	-1.098, 0.014	0.056	-2.440, 1.355	94.1%, 0.521	<0.001
Depression							
Overall	15	-0.258	-0.347, -0.170	<0.001	-0.650, 0.083	44.4%, 0.025	0.033
Treatment duration							
6 weeks	6	-0.214	-0.341, -0.087	0.001	-0.644, 0.133	36.1%, 0.015	0.166
8 weeks	9	-0.295	-0.479, -0.111	0.002	-0.800, 0.210	51.4%, 0.039	0.036
Time points of outcome measurement							
Immediately post-intervention	7	-0.347	-0.499, -0.235	<0.001	-0.755, -0.046	28.9%, 0.014	0.208
4-9weeks post-intervention	6	-0.203	-0.333, -0.073	0.002	-0.519, 0.092	22.4%, 0.008	0.266
Fatigue							
Overall	15	-0.399	-0.562, -0.236	<0.001	-0.963, 0.166	68.4%, 0.062	<0.001
Treatment duration							
6 weeks	9	-0.210	-0.309, -0.112	<0.001	-0.426, -0.021	15.8%, 0.005	0.302
8 weeks	6	-0.654	-1.050, -0.258	0.001	-1.842, 0.535	78.4%, 0.173	<0.001
Time points of outcome measurement							
Immediately post-intervention	8	-0.467	-0.743, -0.191	0.001	-1.316, 0.382	77.2%, 0.109	<0.001
4-9weeks post-intervention	6	-0.228	-0.352, -0.105	<0.001	-0.506, 0.025	17.6%, 0.006	0.300
Overall quality of life							
Overall	8	0.262	0.059, 0.465	0.011	-0.311, 0.836	63.5%, 0.048	0.008
Treatment duration							
6 weeks	2	-0.047	-0.208, 0.113	0.564	-1.089, 0.994	0%, 0	0.728
8 weeks	6	0.402	0.247, 0.556	<0.001	0.199, 0.604	0%, 0	0.629
Time points of outcome measurement							
Immediately post-intervention	5	0.328	0.054, 0.602	0.019	-0.442, 1.098	64.5%, 0.057	0.024
4-9weeks post-intervention	3	0.175	-0.207, 0.557	0.369	-1.262, 1.613	71.8%, 0.074	0.029

#### 3.3.2 Depression

15 studies assessed depression outcome. Given the low heterogeneity among the included studies (I^2^ = 44.4%, Tau^2^ = 0.025), a fixed-effects model was applied for analysis. The overall analysis found that MBSR significantly mitigated depressive symptoms compared to the control group (SMD: -0.258, 95% CI: -0.347 to -0.170, 95% PI: -0.650 to 0.083) (**Table 2**; **Figure 2B**). Subgroup analyses further revealed that both the 6-week and 8-week MBSR interventions led to significant reductions in depression (all *p* < 0.05). Moreover, regardless of whether depression was assessed immediately post-intervention or at 4 to 9 weeks post-intervention, patients in the MBSR group exhibited significantly greater improvements in depressive symptoms relative to the control group (all *p* < 0.05) (**Table 2**; **Supplementary Figure 2**).

#### 3.3.3 Fatigue

**Figure 3:**
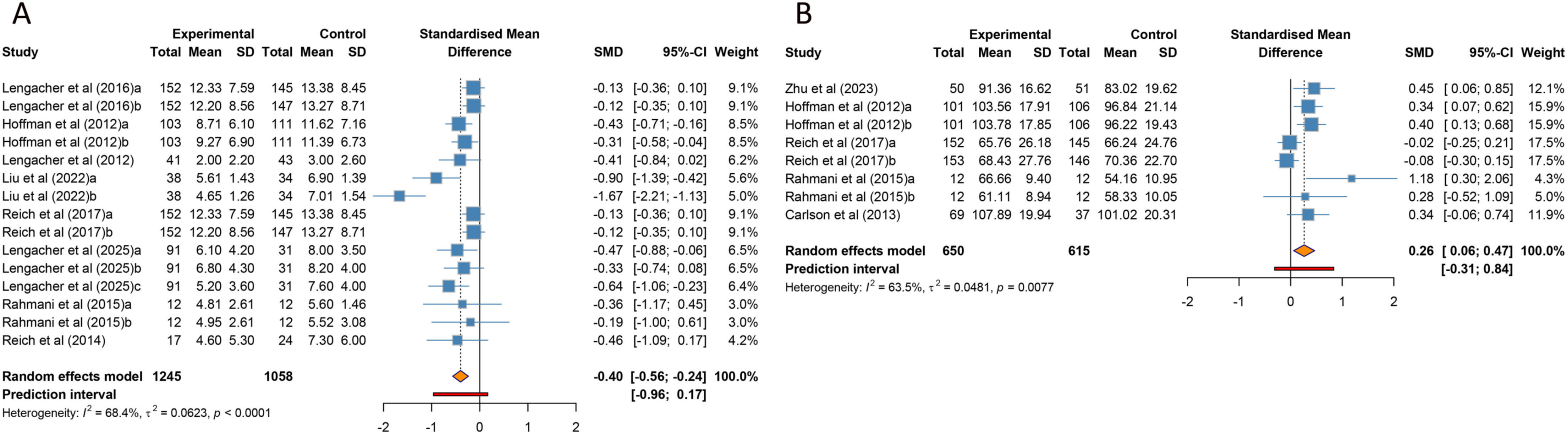
Forest plots of fatigue **(A)** and overall quality of life **(B)** after mindfulness-based stress reduction for breast cancer patients.

Fatigue was assessed as an outcome in 15 studies. Given the marked heterogeneity across the included studies (I^2^ = 68.4%, Tau^2^ = 0.062), a random-effects model was employed for the analysis. The results indicated a significant reduction in fatigue levels among breast cancer patients who received MBSR compared to those in the control group (SMD: -0.399, 95% CI: -0.562 to -0.236, 95% PI: -0.963 to 0.166) (**Table 2**; **Figure 3A**). Subgroup analyses revealed that both the 6-week and 8-week MBSR programs were effective in reducing fatigue (all *p* < 0.05). Furthermore, improvements in fatigue symptoms were observed in the MBSR group irrespective of whether assessments were conducted immediately after the intervention or during follow-up periods ranging from 4 to 9 weeks (all *p* < 0.05) (**Table 2**; **Supplementary Figure 3**).

#### 3.3.4 Overall quality of life

8 studies reported overall QoL. Due to significant heterogeneity among the included studies (I^2^ = 63.5%, Tau^2^ = 0.048), a random-effects model was used for the analysis. The findings revealed that breast cancer patients who participated in MBSR exhibited significantly greater improvements in overall QoL compared to those in the control group (SMD: 0.262, 95% CI: 0.059 to 0.465, 95% PI: -0.311 to 0.836) (**Table 2**; **Figure 3B**). This significance was observed only in the subgroup receiving the 8-week MBSR intervention (SMD: 0.402, 95% CI: 0.247 to 0.556, 95% PI: 0.199 to 0.604) and in the subgroup assessed immediately post-intervention (SMD: 0.328, 95% CI: 0.054 to 0.602, 95% PI: -0.442 to 1.098). In contrast, no differences in overall QoL between the two groups were observed in the subgroup receiving the 6-week MBSR intervention or in the subgroup assessed at 4–9 weeks post-intervention (all *p* > 0.05) (**Table 2**; **Supplementary Figure 4**).

### 3.4 Meta-analysis of the secondary outcomes

#### 3.4.1 Perceived stress

**Figure 4:**
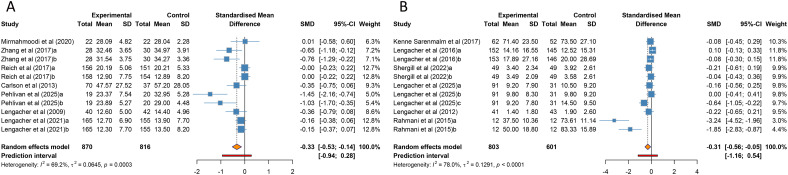
Forest plots of perceived stress **(A)** and pain **(B)** after mindfulness-based stress reduction for breast cancer patients.

11 studies assessed perceived stress. Pooled analysis using a random-effects model (I^2^ = 69.2%, Tau^2^ = 0.065) revealed that patients in the MBSR group showed significantly lower levels of perceived stress compared to the control group (SMD: -0.331, 95% CI: -0.526 to -0.137, 95% PI: -0.939 to 0.276) (**Table 3**; **Figure 4A**). Subgroup analyses based on intervention duration found that patients in the 8-week MBSR intervention group experienced significantly lower levels of perceived stress (SMD: -0.662, 95% CI: -1.030 to -0.293, 95% PI: -1.700 to 0.377), whereas no significant association was observed in the 6-week MBSR intervention group (SMD: -0.097, 95% CI: -0.204 to 0.010, 95% PI: -0.249 to 0.055). Moreover, regardless of whether perceived stress was assessed immediately post-intervention or at 4 to 9 weeks post-intervention, patients in the MBSR group consistently showed significantly lower levels of perceived stress compared to the control group (all *p* < 0.05) (**Table 3**; **Supplementary Figure 5**).

**Table 3:** Pooled effect and subgroup analysis of the secondary outcomes of mindfulness-based stress reduction for breast cancer.

Outcomes and subgroups	Number of studies	Meta-analysis				Heterogeneity	
		SMD/MD	95% CI	p value	95% PI	I^2^, Tau^2^	p value
Perceived stress							
Overall	11	-0.331	-0.526, -0.137	0.001	-0.939, 0.276	69.2%, 0.065	<0.001
6-week treatment duration	5	-0.097	-0.204, 0.010	0.076	-0.249, 0.055	0%, 0	0.496
8-week treatment duration	6	-0.662	-1.030, -0.293	<0.001	-1.700, 0.377	61.7%, 0.128	0.023
Post-intervention immediate assessment	7	-0.335	-0.595, -0.075	0.012	-1.081, 0.411	68.7%, 0.075	0.004
Post-intervention assessment at 4–9 weeks	4	-0.365	-0.726, -0.005	0.047	-1.501, 0.771	77.3%, 0.094	0.004
Pain							
Overall	11	-0.308	-0.563, -0.054	0.018	-1.159, 0.543	78.0%, 0.129	<0.001
6-week treatment duration	6	-0.128	-0.320, 0.065	0.194	-0.625, 0.369	50.4%, 0.028	0.073
8-week treatment duration	5	-0.820	-1.508, -0.132	0.020	-2.994, 1.354	87.9%, 0.490	<0.001
Post-intervention immediate assessment	4	-0.534	-1.185, 0.117	0.108	-2.698, 1.630	88.7%, 0.352	<0.001
Post-intervention assessment at 4–9 weeks	6	-0.280	-0.589, 0.030	0.076	-1.186, 0.627	72.3%, 0.100	0.003
Mental health							
Overall	4	0.100	-0.099, 0.298	0.325	-0.223, 0.422	0%, 0	0.775
6-week treatment duration	1	0.277	-0.158, 0.712	0.212			
8-week treatment duration	3	0.053	-0.170, 0.276	0.641	-0.437, 0.543	0%, 0	0.859
Post-intervention immediate assessment	1	0.277	-0.158, 0.712	0.212			
Post-intervention assessment at 4–9 weeks	2	0.015	-0.255, 0.284	0.916	-1.735, 1.764	0%, 0	0.813
Posttraumatic growth							
Overall	4	7.408	6.002, 8.814	<0.001	2.870, 12.251	31.7%, 1.204	0.222
8-week treatment duration	4	7.408	6.002, 8.814	<0.001	2.870, 12.251	31.7%, 1.204	0.222
Post-intervention immediate assessment	2	6.254	4.259, 8.250	<0.001	-6.682, 19.191	0%, 0	0.673
Post-intervention assessment at 4–9 weeks	2	8.545	6.564, 10.526	<0.001	-29.333, 48.085	39.9%, 4.814	0.197
Fear of recurrence							
Overall	3	-0.816	-2.053, 0.421	0.196	-5.389, 3.757	61.4%, 0.731	0.075
6-week treatment duration	3	-0.816	-2.053, 0.421	0.196	-5.389, 3.757	61.4%, 0.731	0.075
Post-intervention immediate assessment	2	-1.128	-3.300, 1.044	0.309	-23.752, 21.496	78.8%, 1.942	0.030
Post-intervention assessment at 4–9 weeks	1	-0.360	-1.581, 0.861	0.563			
General health							
Overall	4	0.854	-1.056, 2.764	0.381	-2.247, 3.955	0%, 0	0.768
6-week treatment duration	3	1.074	-0.908, 3.056	0.288	-3.277, 5.425	0%, 0	0.788
8-week treatment duration	1	-2.000	-9.140, 5.140	0.583			
Post-intervention immediate assessment	2	1.106	-1.059, 3.270	0.317	-12.927, 15.138	0%, 0	0.493
Post-intervention assessment at 4–9 weeks	2	-0.030	-4.088, 4.028	0.988	-26.340, 26.279	0%, 0	0.511
Total mood disturbance							
Overall	5	-7.955	-15.805, -0.105	0.047	-32.158, 16.248	77.5%, 59.948	0.001
8-week treatment duration	5	-7.955	-15.805, -0.105	0.047	-32.158, 16.248	77.5%, 59.948	0.001
Post-intervention immediate assessment	2	-14.866	-22.527, -7.205	<0.001	-76.491, 47.098	14.2%, 5.4672	0.280
Post-intervention assessment at 4–9 weeks	2	-6.510	-23.746, 10.725	0.459	-192.909, 179.888	89.0%, 137.874	0.003
Sleep quality							
Overall	6	-1.018	-2.263, 0.227	0.109	-4.960, 2.924	82.4%, 1.9482	<0.001
6-week treatment duration	4	-0.189	-0.789, 0.410	0.536	-1.163, 0.784	0%, 0	0.925
8-week treatment duration	2	-2.520	-5.587, 0.547	0.107	-35.854, 30.814	90.5%, 4.433	0.001
Post-intervention immediate assessment	3	-1.593	-4.128, 0.943	0.218	-12.316, 9.131	91.1%, 4.538	<0.001
Post-intervention assessment at 4–9 weeks	2	-0.083	-0.924, 0.758	0.846	-5.535, 5.369	0%, 0	0.569

#### 3.4.2 Pain

11 studies reported pain outcome in breast cancer patients following the intervention. Pooled analyses using a random-effects model (I^2^ = 78.0%, Tau^2^ = 0.129) showed that MBSR significantly reduced pain compared to the control group (SMD: -0.308, 95% CI: -0.563 to -0.054, 95% PI: -1.159 to 0.543) (**Table 3**; **Figure 4B**). This significant reduction was observed only in the subgroup receiving the 8-week MBSR intervention (SMD: -0.820, 95% CI: -1.508 to -0.132, 95% PI: -2.994 to 1.354). No significant association was found in the remaining subgroups (all *p* > 0.05) (**Table 3**; **Supplementary Figure 6**).

#### 3.4.3 Mental health and posttraumatic growth

**Figure 5:**
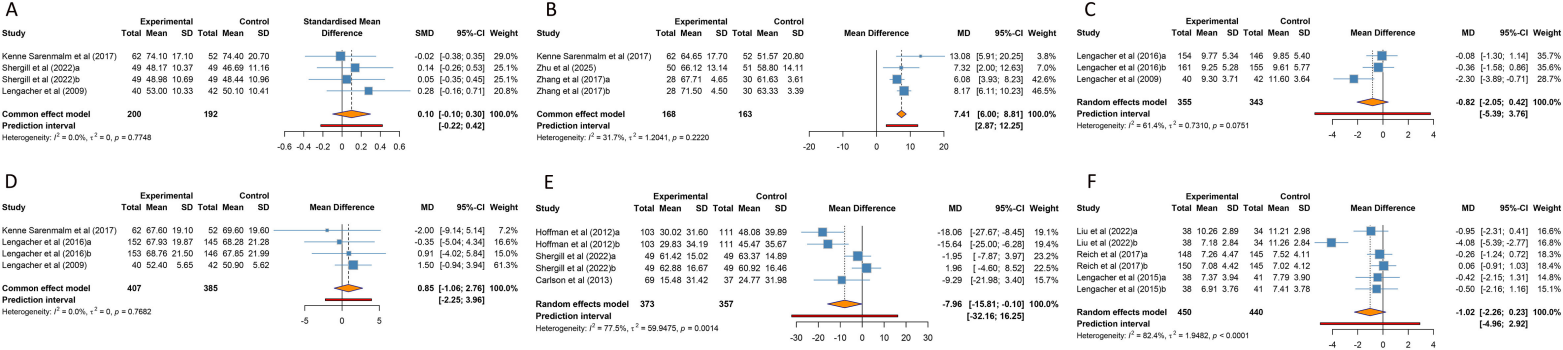
Forest plots of mental health **(A)**, posttraumatic growth **(B)**, fear of recurrence **(C)**, general health **(D)**, total mood disturbance **(E)**, and sleep quality **(F)** after mindfulness-based stress reduction for breast cancer patients.

Mental health outcome was evaluated in 4 studies. Meta-analysis using a fixed-effects model (I^2^ = 0%, Tau^2^ = 0) found no significant difference in mental health status between the MBSR group and the control group (SMD: 0.100, 95% CI: -0.099 to 0.298, 95% PI: -0.223 to 0.422) (**Table 3**; **Figure 5A**). Similarly, subgroup analyses stratified by intervention duration and post-intervention assessment timing yielded no significant findings (all *p* > 0.05) (**Table 3**; **Supplementary Figure 7**).

4 studies reported posttraumatic growth in breast cancer patients. Pooled analyses using a fixed-effects model (I^2^ = 31.7%, Tau^2^ = 1.204) revealed that posttraumatic growth levels were significantly higher in breast cancer patients who underwent MBSR compared with the control group (MD: 7.408, 95% CI: 6.002 to 8.814, 95% PI: 2.870 to 12.251) (**Table 3**; **Figure 5B**). As all 4 included studies implemented an 8-week MBSR intervention, subgroup analyses based on intervention duration were consistent with the overall findings (MD: 7.408, 95% CI: 6.002 to 8.814, 95% PI: 2.870 to 12.251). Additionally, subgroup analyses based on post-intervention assessment time points consistently demonstrated higher posttraumatic growth in the MBSR group relative to the control group (all *p* < 0.05) (**Table 3**; **Supplementary Figure 8**).

#### 3.4.4 Fear of recurrence and general health

Fear of cancer recurrence was evaluated in 3 studies. Meta-analysis using a random-effects model (I^2^ = 61.4%, Tau^2^ = 0.731) showed no significant difference in fear of cancer recurrence between participants receiving MBSR and those in control group (MD: -0.816, 95% CI: -2.053 to 0.421, 95% PI: -5.389 to 3.757) (**Table 3**; **Figure 5C**). Subgroup analyses based on intervention duration and post-intervention assessment time points similarly revealed no significant results (all *p* > 0.05) (**Table 3**; **Supplementary Figure 9**).

General health outcome was examined in 4 studies. Pooled analyses using a fixed-effects model (I^2^ = 0%, Tau^2^ = 0) indicated no significant difference in general health status between the MBSR group and the control group (MD: 0.854, 95% CI: -1.056 to 2.764, 95% PI: -2.247 to 3.955) (**Table 3**; **Figure 5D**). Subgroup analyses based on intervention duration and post-intervention assessment time points also revealed no significant results (all *p* > 0.05) (**Table 3**; **Supplementary Figure 10**).

#### 3.4.5 Total mood disturbance and sleep quality

5 studies assessed total mood disturbance in breast cancer patients. Meta-analysis using a random-effects model (I^2^ = 77.5%, Tau^2^ = 59.948) showed that MBSR significantly mitigated total mood disturbance compared with the control group (MD: -7.955, 95% CI: -15.805 to -0.105, 95% PI: -32.158 to 16.248) (**Table 3**; **Figure 5E**). As all 5 included studies implemented an 8-week MBSR intervention, subgroup analyses based on intervention duration were consistent with the overall findings (MD: -7.955, 95% CI: -15.805 to -0.105, 95% PI: -32.158 to 16.248). Additionally, measurements taken immediately after the intervention showed significant improvement in total mood disturbance in the MBSR group compared to the control group (MD: -14.866, 95% CI: -22.527 to -7.205, 95% PI: -76.491 to 47.098). However, at 4 to 9 weeks post-intervention, no significant difference in total mood disturbance was observed between the MBSR and control groups (*p* > 0.05) (**Table 3**; **Supplementary Figure 11**).

6 studies reported sleep quality in breast cancer patients. Pooled analysis using a random-effects model (I^2^ = 82.4%, Tau^2^ = 1.9482) indicated no significant difference in sleep quality between the MBSR group and the control group (MD: -1.018, 95% CI: -2.263 to 0.227, 95% PI: -4.960 to 2.924) (**Table 3**; **Figure 5F**). Subgroup analyses based on intervention duration and post-intervention assessment time points similarly revealed no significant results (all *p* > 0.05) (**Table 3**; **Supplementary Figure 12**).

### 3.5 Trial sequential analysis results

**Figure 6:**
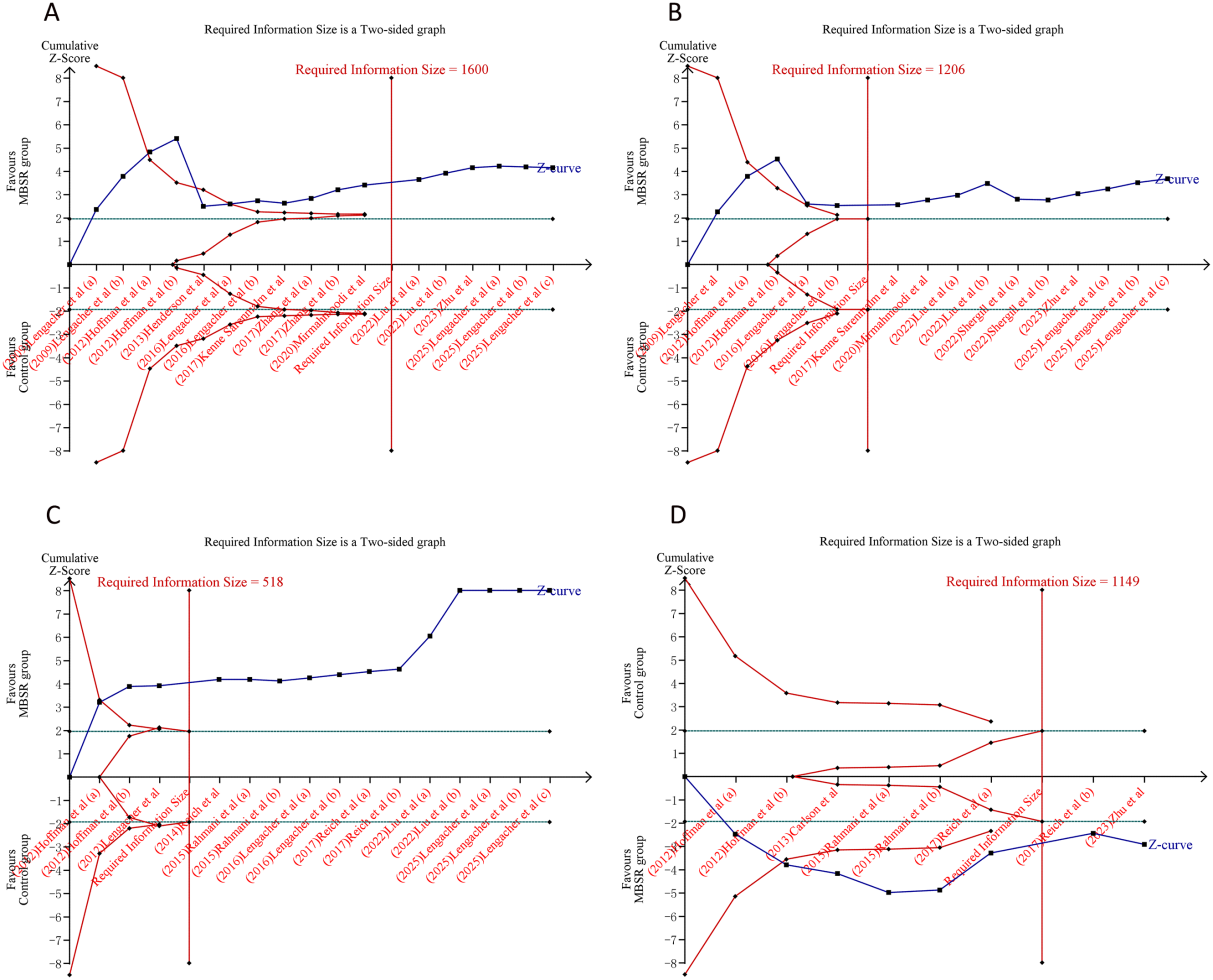
Trial sequential analysis of the primary outcomes after mindfulness-based stress reduction for breast cancer patients. **(A)** Anxiety; **(B)** Depression; **(C)** Fatigue; **(D)** Overall quality of life. Uppermost and lowermost red curves represent trial sequential monitoring boundary lines for benefit and harm, respectively. Inner red lines represent the futility boundary. Blue line represents evolution of cumulative Z-score. Horizontal green lines represent the conventional boundaries for statistical significance. Cumulative Z-curve crossing the trial sequential monitoring boundary or the RIS boundary provides firm evidence of effect.

We conducted TSA for all 12 outcomes included in the meta-analysis. The calculated results showed that for primary outcomes (anxiety, depression, fatigue, and overall QoL), the cumulative Z-curves crossed the trial sequential monitoring boundaries or the RIS boundaries, signifying that the pooled results for these outcomes are robust and definitive, with no need for further studies (**Figure 6**). Among the secondary outcomes, the cumulative Z-curves for perceived stress, pain, and total mood disturbance crossed the monitoring or RIS boundaries. However, the cumulative Z-curves for mental health, fear of recurrence, general health, and sleep quality failed to reach either the monitoring boundaries or the RIS thresholds, suggesting that the evidence for these outcomes remains inconclusive and may be prone to false-positive results (**Supplementary Figure 13**). Regarding posttraumatic growth, the calculated RIS was smaller than the sample size of any individual study included in the meta-analysis, preventing the generation of a TSA graph for this outcome.

### 3.6 Sensitivity analysis, publication bias, and risk of bias due to missing results

Sensitivity analyses and assessments of risk of bias due to missing results were conducted for outcomes with at least 10 studies. Leave-one-out sensitivity analyses showed that removal of any single study did not materially change the pooled estimates for anxiety, depression, fatigue, or perceived stress, supporting the robustness of these findings. In contrast, exclusion of the study by Rahmani et al. influenced the pooled estimate for pain, indicating that this result should be interpreted cautiously (**Supplementary Figure 14**).

For each eligible synthesis, funnel plots were inspected and Begg’s and Egger’s tests were performed, with the trim-and-fill method used to explore the potential effect of missing studies. Potential publication bias was detected for anxiety, fatigue, pain, and perceived stress (all *p* < 0.05), indicating some concerns regarding risk of bias due to missing results. However, trim-and-fill analyses showed that the direction and statistical significance of these pooled effects remained unchanged. No evidence of publication bias was found for depression (Begg’s and Egger’s tests, all *p* > 0.05). For syntheses with fewer than 10 studies, including overall QoL, mental health, posttraumatic growth, fear of recurrence, general health, total mood disturbance, and sleep quality, the risk of bias due to missing results could not be reliably assessed because of the limited number of studies. The corresponding trim-and-fill funnel plots are shown in **Supplementary Figure 15**.

## 4 Discussion

Patients diagnosed with breast cancer frequently face a range of complications, including fatigue, pain, nausea, and neurocognitive dysfunction, which substantially compromise their psychological health, overall well-being, and QoL. These multifaceted complications often exacerbate emotional distress, contributing to elevated levels of anxiety and depression (54). MBSR has emerged as a promising intervention to enhance QoL by fostering emotional resilience, lowering stress, and improving psychological outcomes (55). Our study, synthesizing findings from previous and the latest RCTs, concluded that MBSR significantly alleviates anxiety, depression, and fatigue while improving overall QoL in patients with breast cancer, consistent with prior research (21, 22, 26). Secondary results further indicated that MBSR contributes to reductions in perceived stress and pain, while supporting posttraumatic growth and alleviating total mood disturbance. Subgroup analyses suggested that the 8-week MBSR protocol yields more benefits for breast cancer patients compared to the 6-week program. Importantly, the improvements in depression, fatigue, perceived stress, and posttraumatic growth remained significant during the 4–9 weeks post-intervention, underscoring the sustained therapeutic benefits of MBSR.

Anxiety and depression represent prevalent psychological problems faced by breast cancer patients undergoing early stages of chemotherapy (56). Research indicates that these negative emotional states, including anxiety and depression, can lead to physical discomfort and significantly diminish the patient’s QoL (57). Specifically, neuroimaging studies have revealed increased activity in the left prefrontal cortex following MBSR training, accompanied by structural changes such as enhanced cortical thickness in regions including the anterior cingulate cortex, insula, and hippocampus. Concurrently, reductions in gray matter density within the amygdala have been observed, suggesting a neurobiological basis for improved emotional regulation. These alterations are closely linked to enhanced emotional processing and regulation, enabling individuals to better manage stress and negative affect (58). MBSR has been shown to refine the body’s awareness of emotions, pain, and thought patterns. It helps individuals build resilience against internal triggers, adopt a more objective perspective toward negative experiences, and reduce the tendency to engage in overly subjective evaluations. Over time, this practice disrupts habitual negative thought patterns and the emotional distress they cause, leading to a notable improvement in QoL (59). Furthermore, studies conducted by Chayadi et al. and Wu et al. provide additional evidence that MBSR outperforms traditional or standard care in reducing psychological distress, anxiety, and depression in breast cancer patients (60, 61).

Fatigue, perceived stress, pain, posttraumatic growth, and total mood disturbance are critical determinants of QoL in breast cancer patients. Our study confirmed the benefits of MBSR in improving these critical outcomes. Cancer-related fatigue, recognized as one of the most prevalent and distressing symptoms among cancer patients, particularly breast cancer survivors (62). Previous evidence-based research has shown that MBSR plays a significant role in alleviating fatigue among individuals diagnosed with breast cancer. It is regarded as an effective complementary intervention for managing fatigue in clinical settings involving breast cancer patients (26). In addition to fatigue, prior research has validated the role of MBSR in reducing perceived stress levels in breast cancer patients (15, 23), aligning with the results of our investigation. Notably, perceived stress has been identified as a mediating factor influencing the overall effectiveness of mindfulness-based approaches (40, 63), suggesting its pivotal role in the therapeutic process. MBSR has also demonstrated efficacy in alleviating pain associated with breast cancer and its treatment (64, 65). Furthermore, neuroimaging evidence highlights the influence of MBSR on neural mechanisms underlying chronic neuropathic pain caused by breast cancer therapy. These findings include enhanced white matter integrity in key brain regions (66) and diminished activation in areas involved in pain perception and emotional processing in response to pain-related stimuli (67). Painful experiences often lead individuals to search for meaning and derive psychological growth from adversity, a phenomenon termed posttraumatic growth (68, 69). In breast cancer populations, perceived social support has emerged as a critical protective factor closely associated with posttraumatic growth, as evidenced by prior investigations (70–72). Enhanced levels of posttraumatic growth observed following MBSR intervention may partially explain the mechanism by which QoL improves, with increased social support playing a mediating role. Additionally, reductions in total mood disturbance observed following MBSR intervention can be attributed to its ability to alleviate symptoms of anxiety, depression and fatigue, further underscoring its therapeutic benefits in breast cancer care.

Our subgroup analyses revealed that compared with usual care, the 8-week MBSR program yielded substantial benefits in improving anxiety, depression, fatigue, overall QoL, perceived stress, pain, posttraumatic growth, and total mood disturbance. In contrast, the 6-week MBSR intervention showed significant improvements only in anxiety, depression, and fatigue. These findings suggest that the 8-week MBSR intervention may confer more benefits than 6-week program in mitigating the psychological and physical burdens associated with breast cancer. Unlike the 6-week adaptation, the 8-week MBSR program emphasizes the gradual internalization of mindfulness practices, allowing individuals to tailor the process to their unique circumstances. This extended duration supports the transition from initial exposure to deeper integration, ultimately fostering positive psychological outcomes (22). From a mechanistic perspective, mindfulness training prioritizes the cultivation of moment-to-moment awareness that is non-judgmental and non-reactive to both internal and external stimuli. This practice facilitates emotional regulation during distressing situations, reduces cognitive rumination on painful experiences, and alleviates negative emotional states. However, these processes require sufficient time to take effect (73), underscoring the importance of program duration. Moreover, follow-up assessment conducted 4 to 9 weeks post-intervention revealed that the significant benefits of MBSR were limited to improvements in depression, fatigue, perceived stress, and posttraumatic growth, with fewer benefits observed compared to immediate post-intervention assessment. These findings suggest that the long-term therapeutic benefits of MBSR in breast cancer patients remains limited. Future high-quality RCTs focusing on the sustained effects of MBSR are warranted to further validate our findings.

The present analysis did not reveal any notable advantages of MBSR over usual care in enhancing mental health, mitigating fear of recurrence, improving general health and sleep quality. One possible explanation for these findings is the limited number of studies available for these specific outcomes, which may have led to pooled estimates that are inherently imprecise and statistically underpowered. Sleep-related outcomes, in particular, may be confounded by the prevalence of chemotherapy-induced insomnia among breast cancer patients (74), which could obscure potential therapeutic effects of MBSR. Additionally, assessments of mental and general health relied on the widely-used but generic tools, the 36-Item Short Form Health Survey (SF-36) and the 12-Item Short Form Health Survey (SF-12), which might not adequately capture symptoms unique to breast cancer patients. Further studies are required to better delineate the role of MBSR in addressing these critical domains of patient well-being.

This study has several limitations. First, large heterogeneity was observed in many pooled results, stemming from differences in breast cancer stages, variations in the design and duration of MBSR intervention, inconsistencies in the measurement tools used for identical outcomes, and discrepancies in the timing of outcome evaluations. Second, the diverse stages of disease progression among breast cancer patients likely introduced confounding effects, compounded by variability in baseline characteristics and prior treatments, which may have influenced the observed results. The absence of detailed data precluded subgroup analyses to account for these potential confounders. Third, the nature of MBSR intervention and usual care comparator makes double-blinding infeasible, which may have affected the methodological quality of the included RCTs. Fourth, TSA results underscored the need for further investigations with larger sample sizes to enhance the reliability and generalizability of the conclusions, particularly for outcomes with a limited number of included studies.

## 5 Conclusion

In conclusion, our study revealed that MBSR offered significant advantages over usual care in alleviating symptoms of anxiety, depression, and fatigue in patients with breast cancer, while also enhancing their overall QoL and multiple QoL domains. Outcome-specific analyses further showed that MBSR was associated with lower perceived stress, reduced pain, improved posttraumatic growth, and decreased total mood disturbance. By contrast, no significant improvements were observed in mental health status, fear of cancer recurrence, general health, or sleep quality, suggesting that the benefits of MBSR may be more pronounced for emotional distress, fatigue-related symptoms, and adaptive psychological growth than for all assessed health-related outcomes. Importantly, the sustained benefits of MBSR on depression, fatigue, perceived stress, and posttraumatic growth were observed for up to 4–9 weeks following the intervention. These results support MBSR as an effective intervention for improving psychological well-being and QoL in breast cancer patients. Future research should focus on the long-term effects of MBSR and its underlying mechanisms, providing a stronger evidence base for the comprehensive rehabilitation of breast cancer patients.

## Data Availability

The original contributions presented in the study are included in the article/**Supplementary Material**. Further inquiries can be directed to the corresponding author.
