# Supplementary material for: Efficacy of mindfulness-based stress reduction versus usual care in patients with breast cancer: a meta-analysis with trial sequential analysis of randomized controlled trials
Source: Front Oncol. 2026 Jul 29;16:1780224. doi: 10.3389/fonc.2026.1780224 (PMC13461429; doi:10.3389/fonc.2026.1780224)
Supplement: Supplementary file 1 [file DataSheet1.docx]

**PubMed 669**

#1 (mindfulness-based stress reduction) OR (mindfulness based stress reduction) OR (MBSR) OR (mindfulness-based intervention) OR (meditation) OR (relaxation therapy) OR (mind-body therapies)

#2 (breast cancer) OR (breast neoplasm) OR (breast tumor) OR (cancer of breast) OR (breast malignant neoplasm) OR (breast malignant tumor) OR (mammary cancer) OR (human mammary neoplasms) OR (breast carcinoma) OR (human mammary carcinoma)

#3 (randomized controlled trial) OR (controlled clinical trial) OR (randomized) OR (clinical trial)

#4 #1 AND #2 AND #3

**Web of Science 1173**

#1 TS=((mindfulness-based stress reduction) OR (mindfulness based stress reduction) OR (MBSR) OR (mindfulness-based intervention) OR (meditation) OR (relaxation therapy) OR (mind-body therapies))

#2 TS=((breast cancer) OR (breast neoplasm) OR (breast tumor) OR (cancer of breast) OR (breast malignant neoplasm) OR (breast malignant tumor) OR (mammary cancer) OR (human mammary neoplasms) OR (breast carcinoma) OR (human mammary carcinoma))

#3 TS=((randomized controlled trial) OR (controlled clinical trial) OR (randomized) OR (clinical trial))

#4 #1 AND #2 AND #3

**The Cochrane Library 694**

#1 All Text=((mindfulness-based stress reduction) OR (mindfulness based stress reduction) OR (MBSR) OR (mindfulness-based intervention) OR (meditation) OR (relaxation therapy) OR (mind-body therapies))

#2 All Text=((breast cancer) OR (breast neoplasm) OR (breast tumor) OR (cancer of breast) OR (breast malignant neoplasm) OR (breast malignant tumor) OR (mammary cancer) OR (human mammary neoplasms) OR (breast carcinoma) OR (human mammary carcinoma))

#3 All Text=((randomized controlled trial) OR (controlled clinical trial) OR (randomized) OR (clinical trial))

#4 #1 AND #2 AND #3

**Embase 1509**

#1 'mindfulness-based stress reduction'/exp OR 'mindfulness based stress reduction'/exp OR mbsr OR 'mindfulness-based intervention'/exp OR 'meditation'/exp OR 'relaxation therapy'/exp OR 'mind-body therapies'/exp

#2 'breast cancer'/exp OR 'breast neoplasm'/exp OR 'breast tumor'/exp OR 'cancer of breast' OR 'breast malignant neoplasm' OR 'breast malignant tumor' OR 'mammary cancer'/exp OR 'human mammary neoplasms' OR 'breast carcinoma'/exp OR 'human mammary carcinoma'/exp

#3 'randomized controlled trial'/exp OR 'controlled clinical trial'/exp OR randomized OR 'clinical trial'/exp

#4 #1 AND #2 AND #3
