# Supplementary material for: Efficacy of mindfulness-based stress reduction versus usual care in patients with breast cancer: a meta-analysis with trial sequential analysis of randomized controlled trials
Source: Front Oncol. 2026 Jul 29;16:1780224. doi: 10.3389/fonc.2026.1780224 (PMC13461429; doi:10.3389/fonc.2026.1780224)
Supplement: Supplementary file 2 [file DataSheet2.docx]

**FIGURE S1** Subgroup analysis of anxiety of mindfulness-based stress reduction treatment for breast cancer. (A) Subgroup = 6 weeks; (B) Subgroup = 8 weeks; (C) Subgroup = Immediately post-intervention; (D) Subgroup = 4-9weeks post-intervention.

**FIGURE S2** Subgroup analysis of depression of mindfulness-based stress reduction treatment for breast cancer. (A) Subgroup = 6 weeks; (B) Subgroup = 8 weeks; (C) Subgroup = Immediately post-intervention; (D) Subgroup = 4-9weeks post-intervention.

**FIGURE S3** Subgroup analysis of fatigue of mindfulness-based stress reduction treatment for breast cancer. (A) Subgroup = 6 weeks; (B) Subgroup = 8 weeks; (C) Subgroup = Immediately post-intervention; (D) Subgroup = 4-9weeks post-intervention.

**FIGURE S4** Subgroup analysis for overall quality of life after mindfulness-based stress reduction treatment for breast cancer. (A) Subgroup = 6 weeks; (B) Subgroup = 8 weeks; (C) Subgroup = Immediately post-intervention; (D) Subgroup = 4-9weeks post-intervention.

**FIGURE S5** Subgroup analysis for perceived stress after mindfulness-based stress reduction treatment for breast cancer. (A) Subgroup = 6 weeks; (B) Subgroup = 8 weeks; (C) Subgroup = Immediately post-intervention; (D) Subgroup = 4-9weeks post-intervention.

**FIGURE S6** Subgroup analysis for pain after mindfulness-based stress reduction treatment for breast cancer. (A) Subgroup = 6 weeks; (B) Subgroup = 8 weeks; (C) Subgroup = Immediately post-intervention; (D) Subgroup = 4-9weeks post-intervention.

**FIGURE S7** Subgroup analysis for mental health after mindfulness-based stress reduction treatment for breast cancer. (A) Subgroup = 6 weeks; (B) Subgroup = 8 weeks; (C) Subgroup = Immediately post-intervention; (D) Subgroup = 4-9weeks post-intervention.

**FIGURE S8** Subgroup analysis for posttraumatic growth after mindfulness-based stress reduction treatment for breast cancer. (A) Subgroup = 8 weeks; (B) Subgroup = Immediately post-intervention; (C) Subgroup = 4-9weeks post-intervention.

**FIGURE S9** Subgroup analysis for fear of recurrence after mindfulness-based stress reduction treatment for breast cancer. (A) Subgroup = 6 weeks; (B) Subgroup = Immediately post-intervention; (C) Subgroup = 4-9weeks post-intervention.

**FIGURE S10** Subgroup analysis for general health after mindfulness-based stress reduction treatment for breast cancer. (A) Subgroup = 6 weeks; (B) Subgroup = 8 weeks; (C) Subgroup = Immediately post-intervention; (D) Subgroup = 4-9weeks post-intervention.

**FIGURE S11** Subgroup analysis for total mood disturbance after mindfulness-based stress reduction treatment for breast cancer. (A) Subgroup = 8 weeks; (B) Subgroup = Immediately post-intervention; (C) Subgroup = 4-9weeks post-intervention.

**FIGURE S12** Subgroup analysis for sleep quality after mindfulness-based stress reduction treatment for breast cancer. (A) Subgroup = 6 weeks; (B) Subgroup = 8 weeks; (C) Subgroup = Immediately post-intervention; (D) Subgroup = 4-9weeks post-intervention.

**FIGURE S13** Trial sequential analysis of the secondary outcomes after mindfulness-based stress reduction treatment for breast cancer. (A) Perceived stress; (B) Pain; (C) Mental health; (D) Fear of recurrence; (E) General health; (F) Total mood disturbance; (G) Sleep quality. Uppermost and lowermost red curves represent trial sequential monitoring boundary lines for benefit and harm, respectively. Inner red lines represent the futility boundary. Blue line represents evolution of cumulative Z-score. Horizontal green lines represent the conventional boundaries for statistical significance. Cumulative Z-curve crossing the trial sequential monitoring boundary or the RIS boundary provides firm evidence of effect.

**FIGURE S14** Sensitivity analysis for the outcomes with ≥ 10 included studies. (A) Anxiety; (B) Depression; (C) Fatigue; (D) Perceived stress; (E) Pain.

**FIGURE S15** The trim-and-fill funnel plots of the outcomes with ≥ 10 included studies. (A) Anxiety; (B) Depression; (C) Fatigue; (D) Perceived stress; (E) Pain.
