# Supplementary material for: Efficacy of mindfulness-based stress reduction versus usual care in patients with breast cancer: a meta-analysis with trial sequential analysis of randomized controlled trials
Source: Front Oncol. 2026 Jul 29;16:1780224. doi: 10.3389/fonc.2026.1780224 (PMC13461429; doi:10.3389/fonc.2026.1780224)
Supplement: Supplementary file 3 [file Table1.docx]

| **TABLE S1** The scales used to measure the primary and secondary outcomes. | |
| --- | --- |
| Outcomes | Scales |
| Anxiety | Self-Rating Anxiety Scale (SAS), Beck Anxiety Inventory (BAI), Hospital Anxiety and Depression Scale (HADS), State-Trait Anxiety Inventory (STAI), Profile of Mood States (POMS), Symptom Checklist-90-Revised (SCL-90), Patient-Reported Outcomes Measurement Information System (PROMIS) |
| Depression | Self-Rating Depression Scale (SDS), Beck Depression Inventory-II (BDI-II), HADS, Center for Epidemiologic Studies Depression Scale (CES-D), POMS, Patient Health Questionnaire-9 (PHQ-9), PROMIS |
| Fatigue | Fatigue Symptom Inventory (FSI), POMS, M.D. Anderson Symptom Inventory (MDASI), Brief Fatigue Inventory (BFI), PROMIS, Fatigue Severity Scale (FSS) |
| Overall quality of life | Functional Assessment of Cancer Therapy-Breast (FACT-B), Short-Form (36) Health Survey (SF-36), European Organization for Research and Treatment of Cancer Quality of Life Questionnaire-Core 30 (EORTC QLQ-C30) |
| Perceived stress | Perceived Stress Scale (PSS), Calgary Symptoms of Stress Inventory (C-SOSI) |
| Pain | SF-36, Brief Pain Inventory (BPI), PROMIS, MDASI, EORTC QLQ-C30 |
| Mental health | SF-36, SF-12 |
| Posttraumatic growth | Posttraumatic Growth Inventory (PTGI) |
| Fear of recurrence | Concerns About Recurrence Scale (CARS) |
| General health | Medical Outcomes Study Short Form (MOS SF-36) |
| Total mood disturbance | POMS |
| Sleep quality | Pittsburgh Sleep Quality Index (PSQI) |

| **TABLE S2** MBSR intervention components and delivery methods in the included studies. | |
| --- | --- |
| Study ID | Detailed description of MBSR |
| Zhu et al (2023) | The MBSR intervention consisted of an 8-week group-based program delivered once weekly, with each session lasting 2 hours. Participants were divided into four groups of 13 patients each. Each session included didactic instruction/lectures, mindfulness practice, and experience sharing or group discussion. Core MBSR components included mindful breathing, meditation, body scan, and yoga, following protocols guided by Jon Kabat-Zinn and Bob Stahl. Sessions were delivered interactively in the breast surgery department by researchers trained in MBSR. Participants were encouraged to practice mindfulness at home 6 days per week for at least 30 minutes per day, supported by mindfulness audio recordings and practice materials. Family members were encouraged to accompany or participate in the training. Daily practice was monitored by an independent researcher, who also answered practice-related questions. Additional supervision and guidance were provided by telephone during home practice, and participants who missed sessions were offered attendance in another group or individualized home-based guidance. |
| Mirmahmoodi et al (2020) | **Session 1:** The study purpose and an overview of the sessions were explained. Participants were then asked to eat a raisin with full sensory awareness and practiced body scan meditation for 30 minutes, directing their attention to different parts of the body. Session 2: Participants discussed their home practice of body scan meditation, including obstacles such as restlessness and mind wandering, possible solutions such as non-judgment and letting go of intrusive thoughts, and the difference between thoughts and feelings. Participants then practiced sitting meditation. Session 3: Participants practiced seeing and listening in a non-judgmental manner for 2 minutes, followed by sitting meditation and mindful breathing with attention to physical sensations. Session 4: Participants focused on bodily sounds and thoughts, discussed stress responses, reactions to difficult situations, and alternative attitudes and behaviors, and practiced mindful walking. Session 5: Participants practiced sitting meditation and were introduced to the second series of mindful body movements and mountain meditation. Session 6: Participants discussed their home practice with one another. The session then focused on the theme that the content of thoughts is not always real. Thereafter, four types of meditation were practiced over 1 hour. Session 7: Participants practiced 4D meditation and awareness of the present moment. The main theme of this session was “What is the best way to take care of myself?” Participants then identified pleasant and unpleasant events and planned an enjoyable activity. Session 8: Participants applied what they had learned. The session began with body scan meditation, followed by a discussion of obstacles to meditation. |
| Kenne Sarenmalm et al (2017) | Participants in the MBSR group received a standardized, group-based 8-week MBSR course delivered once weekly for approximately 2 hours per session, led by a certified MBSR instructor. The intervention combined self-instructed mindfulness training with instructor-led weekly group sessions. Group sessions focused on participants’ experiences of mindfulness and included gentle meditation and yoga training. Participants were assigned home practice consisting of 20-minute sessions, 6 days per week. Intervention materials included a 20-page introduction to mindfulness training, a CD with meditation exercises, an 8-week training program, and a diary for recording time spent on mindfulness practice and reflections on meditation exercises. Written and oral instructions were provided on how to use the materials, CD, and diary. All participants also received standard breast cancer follow-up care according to national and local guidelines. |
| Lengacher et al (2016) | Participants received a breast cancer-adapted MBSR intervention [MBSR(BC)] consisting of 2-hour sessions once weekly for 6 weeks. The program was delivered by a clinical psychologist trained in MBSR and was adapted from the standard 8-week Kabat-Zinn MBSR program to address the specific needs, concerns, and symptoms of breast cancer survivors. Intervention components included educational materials, practice of four meditative techniques, namely sitting meditation, walking meditation, body scan, and gentle Hatha yoga, and group processes addressing barriers to meditation practice, application of mindfulness in daily situations, and supportive interaction among group members. Participants were also taught informal mindfulness techniques for integration into daily activities. Training manuals and CDs were provided to support home practice. Compliance was assessed by class attendance, diary completion, and minutes of practice. Adequate compliance was defined as attendance at at least 75% of sessions and completion of at least 75% of assigned homework, corresponding to 15-45 minutes of daily practice. |
| Zhu et al (2025) | The experimental group received an 8-week MBSR program based on materials by Jon Kabat-Zinn and Bob Stahl. The intervention included mindfulness breathing, mindfulness meditation, body scan, and mindfulness yoga. Participants were instructed to practice mindfulness training at home 6 days per week for at least 30 minutes per day, following the weekly course content. Mindfulness training audio recordings and practice materials were provided to guide daily practice. Family members were encouraged to accompany patients or participate in the training. Researchers checked participants’ mindfulness practice daily and answered practice-related questions. During home practice, researchers provided telephone supervision and guidance. Participants who were absent from sessions were allowed to attend other courses during the same period or received individualized guidance from the researcher. |
| Hoffman et al (2012) | Participants received a standardized 8-week MBSR program closely based on the Kabat-Zinn model. The program consisted of eight weekly classes, each lasting 2 hours, except for the first and final classes, which lasted 2.25 hours, plus one 6-hour mindfulness day in week 6. Each group included 12-20 participants. Formal mindfulness practices included body scan, gentle and appropriate lying and standing yoga-based stretches, and sitting meditation. Sessions also incorporated group discussion, didactic teaching, and home practice related to perceptions of and reactions to life events, stress physiology, and mindfulness in communication and everyday life. Home practice was supported by four 45-minute CDs containing formal mindfulness practices and a manual. Participants were instructed to practice for 40-45 minutes per day, 6 or 7 days per week, and recorded the duration and amount of formal home practice using weekly record sheets. The intervention was delivered by a clinician-researcher qualified as an MBSR instructor at the University of Massachusetts Center for Mindfulness, with clinical supervision provided by a senior teacher trainer. Participants could contact the clinician-researcher during or after the study period for study-related concerns. |
| Shergill et al (2022) | The intervention followed the standard MBSR format, with minor facilitator-specific modifications, such as variation in mindful movement practices and greater emphasis on clarifying personal values. It consisted of eight weekly 2.5-hour group sessions and one full-day retreat of approximately 6 hours held halfway through the course on a weekend. Each group included approximately 8-12 participants. Courses were coordinated by the research team and delivered at the Ottawa Regional Cancer Foundation; when participants could not attend a research-arranged group, they were enrolled in community-based MBSR programs offered by other healthcare providers. Participants could choose a convenient course location and time across the region. Each session included mindfulness meditation practice, discussion of participants’ meditation experiences, and application of mindfulness in daily life. Group discussions addressed themes such as stress, pain, personal attitudes and values, and mindful communication. Sessions were delivered primarily by healthcare professionals, including psychologists and social workers, with formal MBSR certification and at least 5 years of experience delivering group-based MBSR. In two cases, sessions were facilitated or co-facilitated by mindfulness-trained postgraduate instructors under supervision from an experienced MBSR healthcare provider. Waitlist participants were offered MBSR after the waiting period. |
| Lengacher et al (2009) | Participants received a breast cancer-adapted MBSR intervention [MBSR(BC)] consisting of weekly 2-hour group sessions over 6 weeks. The program was adapted from Kabat-Zinn’s original 8-week MBSR program to address the health status and symptom burden of breast cancer survivors, including emotional and psychological symptoms such as anxiety, depression, and fear of recurrence, as well as physical symptoms such as pain and sleep disturbance. Class sizes ranged from 4 to 8 participants, and seven groups completed the intervention over 15 months. All sessions were standardized and followed a training manual developed to ensure consistency. The intervention was delivered by a single psychologist certified and trained in MBSR. An independent observer monitored weekly sessions for consistency by recording the timing of intervention activities, and session quality was assessed using qualitative post-observation reports. Core intervention components included educational materials on relaxation, meditation, and the mind-body connection; group-based practice of meditation and yoga; homework assignments; and group discussion of barriers to meditation practice, application of mindfulness in daily life, and group support. Mindfulness practices included sitting meditation, body scan, walking meditation, visualization, awareness of breathing, yoga postures, mountain and/or lake meditation, and awareness of bodily sensations, sounds, thoughts, and feelings. Participants received a training manual and four audiotapes to support home practice of sitting meditation, body scan, walking meditation, and gentle yoga. The manual included weekly objectives, exercises, program content, and a daily diary for recording homework practice. During week 1, participants received an overview of the intervention and learned initial meditation and body scan procedures. Week 2 introduced visualization and sitting meditation with awareness of breathing as the primary focus. Weeks 3 and 4 addressed reactions to pleasant and unpleasant events, body scan responses to stress, physiological correlates of stress, and yoga postures. Week 5 focused on expanding awareness and modifying stress-inducing patterns, including mountain and/or lake meditation and awareness of bodily sensations, sounds, thoughts, and feelings. Week 6 encouraged participants to internalize the practices and develop a lifelong mindfulness practice. Participants were asked to formally practice sitting meditation, walking meditation, body scan, and yoga for 15-45 minutes per day, 6 days per week, with practice time increasing as they gained experience. They were also asked to practice informal mindfulness for 15-45 minutes per day. Daily practice was recorded in a diary. Compliance was defined a priori as attendance at at least 75% of MBSR(BC) sessions and completion of at least 75% of assigned homework, based on a minimum of 45 minutes of practice per day; total practice time across the 6-week program was also assessed. |
| Henderson et al (2013) | Women randomized to the MBSR group received a group-based MBSR intervention delivered at a single study site. The intervention included an introductory meeting for BRIDGES-only participants, followed by eight weekly group sessions lasting 2.5-3.5 hours each, with groups of 25-30 women. An additional 7.5-hour intensive retreat session was held during week 6. After completion of the 8-week MBSR program, participants attended three additional 2-hour monthly support sessions to discuss practice-related issues and provide ongoing support. The MBSR program was delivered by instructors with master’s or doctoral degrees and long-term meditation practice. The three monthly follow-up sessions were led by a psychiatrist with MBSR internship training and long-term meditation practice. |
| Lengacher et al (2025) | Breast cancer survivors assigned to MBSR(BC) received a 6-week breast cancer-adapted MBSR program, modified from Kabat-Zinn’s standard 8-week program. The intervention consisted of six weekly 2-hour group sessions conducted by a psychologist and a social worker trained in MBSR. The program aimed to enhance mindfulness-based cognitive training in self-regulation of awareness, attention, and concentration. Core components included educational materials, formal meditation practice, informal mindfulness practice in everyday life, and group supportive interaction. Formal practices included sitting meditation, body scan, gentle Hatha yoga, and walking meditation. Participants practiced mindfulness meditation techniques for 15-45 minutes per day and recorded their practice time. A manual and compact discs were provided to support home practice. Intervention fidelity was monitored using a structured observational method, including time-sequenced evaluation of instructor adherence to the MBSR(BC) protocol by a research assistant for each group, with monthly assessment of adherence to protocol delivery. |
| Zhang et al (2017) | Participants assigned to the MBSR(BC) group received weekly 2-hour group sessions conducted by a psychologist certified and qualified in mindfulness skills. Before the trial, an 8-week pilot feasibility MBSR group was conducted with five hospital patients to allow the instructor to practice delivering the program under clinical supervision and to resolve practical delivery issues; pilot data were not analyzed. After the pilot, six MBSR groups were delivered, with 4-6 participants per group. All sessions were based on Kabat-Zinn’s original 8-week MBSR program and adapted to participants’ conditions. Classes included four basic forms of meditation practice: body scan, walking meditation, gentle yoga, and sitting meditation. Sessions also incorporated group discussion, didactic teaching, and home practice. Participants received an MBSR homework book manual and audio download information before the intervention. They were instructed to complete homework assignments and practice for 40-45 minutes per day, 6 or 7 days per week, and to record homework practice activities in the manual. At the beginning of each weekly session, participants discussed their homework experiences, barriers to meditation practice, and applications of mindfulness in daily life, thereby enhancing group interaction and support. Each class then included instruction and practice of mindfulness components. Participants with study-related concerns could contact the psychologist during or after the study period. |
| Lengacher et al (2012) | Participants received a breast cancer-adapted MBSR intervention [MBSR(BC)] condensed from Kabat-Zinn’s original 8-week program into a 6-week format, with the 1-day 8-hour silent retreat excluded. The program consisted of six weekly 2-hour group sessions delivered by a licensed clinical psychologist trained in MBSR and retained the original content of the standard 8-week program. The intervention aimed to increase awareness of thoughts, feelings, and responses during stressful situations through meditation practice. In addition to standard MBSR content, the program included group support sessions focused on emotional and psychological responses, such as anxiety, depression, and fear of recurrence, and physical symptoms common among women with breast cancer, such as pain and sleep problems. Core components included educational material on relaxation, meditation, and the mind-body connection; meditation practice during weekly group sessions and homework assignments; group discussion of barriers to meditation practice and the application of mindfulness in daily life; and supportive interaction among group members. Formal meditative training included four techniques: sitting meditation, walking meditation, body scan, and gentle Hatha yoga, with attention focused on the breath. Informal mindfulness practice was used to teach participants to integrate mindfulness into everyday life, including awareness of pleasant and unpleasant events, routine activities, and daily experiences. |
| Liu et al (2022) | Patients randomized to the MBSR group received an 8-week MBSR program consisting of weekly group sessions and daily individual practice. The group sessions were delivered by the primary interventionist, together with one nurse, in a large recreational area of the breast oncology department. Participants were required to practice MBSR once daily for 45 minutes throughout the 8-week intervention. Individual practice was conducted independently using audio recordings during both inpatient and outpatient periods and was monitored and guided by a nurse. The MBSR program included eight sessions: breath awareness, seated mindfulness meditation, focusing on one thing, body scanning, compassionate meditation, sounds and thoughts, choiceless awareness, and coming full circle. During hospitalization, daily practice was recommended at approximately 10 a.m. to avoid interference with meals and rest periods. Participants could practice on the ward bed or in the recreational area according to preference, and each patient was provided with a mini speaker to listen to session instructions during daily practice. Daily face-to-face discussions between interventionists and patients were conducted to ensure adherence to program content and objectives and to address practice-related issues. Participants were asked to record daily mindfulness practice in a log, which was checked daily by the interventionists. After discharge, participants were instructed to continue daily practice on schedule, and weekly group sessions were delivered via WeChat to address issues encountered during practice. |
| Reich et al (2017) | Participants received a breast cancer-adapted MBSR intervention [MBSR(BC)] modeled after Jon Kabat-Zinn’s MBSR program and adapted to support stress reduction and symptom management through the self-regulatory process of meditation. The 6-week program consisted of weekly 2-hour sessions delivered by a psychologist trained in MBSR. The intervention included three main components: educational material on relaxation, meditation, the mind-body connection, and healthy lifestyle for survivors; meditation practice during group sessions and homework assignments; and group processes addressing barriers to meditation practice and supportive interaction among group members. Formal meditation techniques included sitting meditation, body scan, gentle Hatha yoga, and walking meditation. Participants were also taught informal techniques for integrating mindfulness into daily life activities. Breast cancer survivors were asked to practice formal and informal meditative techniques for 15-45 minutes per day and to record their practice time in a daily diary. A manual and compact discs were provided to guide home practice. |
| Rahmani et al (2015) | **Session 1:** Introduction to the automatic guidance system and to the use of present-moment awareness of bodily sensations, thoughts, and emotions for stress reduction; raisin-eating exercise; feedback and discussion about the practice; three-minute breathing exercise; assignment for the following week; and distribution of leaflets for Session 1 and meditation CDs. Session 2: Review of the body scan exercise; feedback and discussion about the body scan practice; breathing mindfulness meditation; yoga stretching exercises; and distribution of leaflets for Session 2 and meditation CDs. Session 3: Sitting meditation with awareness of breathing; yoga exercises practiced in the hospital chapel; three-minute breathing exercise; and distribution of leaflets for Session 3 and a videotape of yoga practices. Session 4: Review of the body scan exercise; mindful yoga exercises practiced in the hospital chapel; 5-minute “seeing or hearing” exercise; sitting meditation with awareness of breathing and the body; and distribution of leaflets for Session 4 and meditation CDs. Session 5: Breathing practice; sitting meditation with awareness of breathing, the body, sounds, and thoughts; explanation of stress and identification of participants’ reactions to stress; awareness of pleasant and unpleasant events and their effects on feelings, thoughts, and bodily sensations; mindful yoga exercises; three-minute breathing exercise; and distribution of leaflets. Session 6: Mindful yoga practice; sitting meditation with mindfulness of sounds and thoughts; and distribution of leaflets for Session 6 and videotape No. 4 to participants. Session 7: Mountain meditation; sleep hygiene education; review of exercises from the previous session; preparation of a list of enjoyable activities; and distribution of leaflets for Session 7. Session 8: Body scan exercise; overview and review of the program; examination and discussion of participants’ practice plans; and stone, beads, and marbles meditation. |
| Lengacher et al (2021) | Breast cancer survivors received a breast cancer-adapted MBSR intervention [MBSR(BC)] consisting of six weekly 2-hour group sessions conducted by a psychologist trained in MBSR(BC). The program was adapted by Dr. Lengacher from Jon Kabat-Zinn’s original 8-week MBSR program for breast cancer survivors. Intervention components included educational materials, practice sessions involving formal and informal meditative techniques, and group processes addressing barriers to practice, application of mindfulness in daily situations, and supportive group interaction. Formal meditation techniques included sitting meditation, body scan meditation, gentle Hatha yoga, and walking meditation. Participants were taught to bring awareness to thoughts and emotions associated with symptoms such as pain, anxiety, sleep disturbance, and fear of recurrence, and to separate emotional experiences from sensory experiences. Informal mindfulness techniques involved integrating attention and awareness into daily activities to promote present-moment awareness and self-regulation of attention. Participants received a training manual and five audio CDs to guide home practice of sitting meditation, body scan, gentle yoga, and walking meditation, and recorded their practice time in a diary throughout the 12-week study period. |
| Carlson et al (2013) | Participants received mindfulness-based cancer recovery (MBCR), a cancer-adapted mindfulness intervention rooted in contemplative spiritual traditions and modeled on the MBSR program developed at the Massachusetts Medical Center. The program was modified by Carlson and Speca as MBCR and had been validated in previous studies. MBCR consisted of eight weekly group sessions, each lasting 90 minutes, plus a 6-hour workshop between weeks 6 and 7, providing a total of 18 contact hours. Sessions were led by trained staff with experience facilitating previous MBCR trials. The average study group size was six participants across both study sites; however, participants were integrated into ongoing clinical groups of up to 20 participants with various cancer types. The intervention emphasized mindfulness as conscious, present-moment awareness practiced in an open and nonjudgmental manner. |
| Pehlivan et al (2025) | Participants received an 8-week mindfulness-based yoga and meditation program adapted from standard MBSR and tailored to the needs of breast cancer patients. The “Day of Silence” retreat was omitted because of potential physical burden. Sessions were delivered weekly in two closed groups, with each session lasting 90 minutes. The intervention was led by a researcher who had completed pretrainer training in mindfulness-based yoga, movement, breathing, and meditation practices and obtained a trainer certificate. Core practices included silence meditation, mindful eating, body scan meditation, Hatha/Yin yoga, sitting meditation with mindful breathing, walking meditation, mountain meditation, lake meditation, loving-kindness meditation, and brief mindfulness practices. Session themes included introduction to mindfulness, perception, body awareness, mindful stress management, nonjudgmental acceptance of stress-related thoughts, mindful communication, self-care, and continuation of practice. Group discussions and sharing were included to support integration of mindfulness into daily life. Homework was assigned throughout the 8 weeks, and a researcher’s handbook containing homework and additional information was provided before the first session. Participants attending less than 75% of sessions were excluded; participants could attend the other group on the same day if they missed their scheduled session. |
| Reich et al (2014) | Participants received a 6-week breast cancer-adapted MBSR program [MBSR(BC)] modified from Kabat-Zinn’s original 8-week MBSR program to address emotional/psychological symptoms, including anxiety, depression, and fear of recurrence, and physical symptoms, including fatigue, pain, and sleep disturbance. Weekly 2-hour sessions were delivered by a psychologist trained in MBSR. The program included educational materials and exercises related to meditation practice and the mind-body connection, practice time supported by a CD with verbal guidance for four meditative practices, and group discussion addressing questions and barriers to formal and informal practice. Formal mindfulness practices included sitting meditation, walking meditation, body scan, and gentle Hatha yoga. Informal mindfulness practice focused on integrating mindfulness into everyday life, including awareness of pleasant and unpleasant events and routine activities. Participants received a manual to guide classes and were asked to practice formally and informally for 15-45 minutes daily outside group sessions and to record practice time in a diary. |
| Lengacher et al (2015) | Participants received a breast cancer-adapted MBSR intervention [MBSR(BC)] derived from Jon Kabat-Zinn’s MBSR program. The intervention consisted of six weekly 2-hour sessions designed to promote active stress reduction and symptom management. Core components included educational materials on relaxation, meditation, the mind-body connection, and healthy lifestyles; practice of meditation, yoga, body scan, and walking meditation; and supportive group interaction and discussion. Participants were asked to practice formal and informal meditative techniques for 15-45 minutes per day and to record practice time throughout the 12-week study period. Compact discs and a diary were provided to facilitate home practice and documentation. |
| MBSR, mindfulness-based stress reduction. | |

| **TABLE S3** Quality analysis of the included RCTs by modified Jadad scale. | | | | | | |
| --- | --- | --- | --- | --- | --- | --- |
| Study | Randomization | Randomization concealment | Double blind | Withdrawals and dropouts | Score | Study quality |
| Zhu et al (2023) | 2 | 2 | 0 | 1 | 5 | High |
| Mirmahmoodi et al (2020) | 2 | 2 | 0 | 1 | 5 | High |
| Kenne Sarenmalm et al (2017) | 2 | 2 | 0 | 1 | 5 | High |
| Lengacher et al (2016) | 2 | 2 | 0 | 1 | 5 | High |
| Zhu et al (2025) | 2 | 2 | 0 | 1 | 5 | High |
| Hoffman et al (2012) | 2 | 2 | 0 | 1 | 5 | High |
| Shergill et al (2022) | 2 | 2 | 0 | 1 | 5 | High |
| Lengacher et al (2009) | 2 | 2 | 0 | 1 | 5 | High |
| Henderson et al (2013) | 1 | 1 | 0 | 1 | 3 | Low |
| Zhang et al (2017) | 2 | 2 | 0 | 1 | 5 | High |
| Lengacher et al (2012) | 2 | 2 | 0 | 1 | 5 | High |
| Liu et al (2022) | 2 | 2 | 0 | 1 | 5 | High |
| Reich et al (2017) | 2 | 2 | 0 | 1 | 5 | High |
| Lengacher et al (2025) | 2 | 2 | 0 | 1 | 5 | High |
| Rahmani et al (2015) | 1 | 1 | 0 | 0 | 2 | Low |
| Lengacher et al (2021) | 2 | 2 | 0 | 1 | 5 | High |
| Carlson et al (2013) | 2 | 2 | 0 | 1 | 5 | High |
| Pehlivan et al (2025) | 2 | 2 | 0 | 1 | 5 | High |
| Reich et al (2014) | 1 | 1 | 0 | 0 | 2 | Low |
| Lengacher et al (2015) | 2 | 2 | 0 | 1 | 5 | High |
